# Named Entity Recognition of Diabetes Online Health Community Data Using Multiple Machine Learning Models

**DOI:** 10.3390/bioengineering10060659

**Published:** 2023-05-29

**Authors:** Qian Xu, Yue Zhou, Bolin Liao, Zirui Xin, Wenzhao Xie, Chao Hu, Aijing Luo

**Affiliations:** 1 Second Xiangya Hospital, Central South University, Changsha 410011, China; qian_xu2000@csu.edu.com (Q.X.); zhouyue8@csu.edu.cn (Y.Z.); xinzirui@csu.edu.cn (Z.X.); 2 School of Life Sciences, Central South University, Changsha 410013, China; 3 College of Computer Science and Engineering, Jishou University, Jishou 416000, China; mulinliao8184@163.com; 4 Key Laboratory of Medical Information Research, Central South University, College of Hunan Province, Changsha 410013, China; xie_wenzhao@126.com; 5 Clinical Research Center for Cardiovascular Intelligent Healthcare in Hunan Province, Changsha 410011, China; 6 Big Data Institute, Central South University, Changsha 410011, China; huchao@csu.edu.cn

**Keywords:** diabetes, online healthcare data, named entity recognition, RoBERTa-BiLSTM-CRF, online health community

## Abstract

The rising prevalence of diabetes and the increasing awareness of self-health management have resulted in a surge in diabetes patients seeking health information and emotional support in online health communities. Consequently, there is a vast database of patient consultation information in these online health communities. However, due to the heterogeneity and incompleteness of the content, mining medical information and patient health data from these communities can be a challenge. To address this issue, we built the RoBERTa-BiLSTM-CRF (RBC) model for identifying entities in the online health community of diabetes. We selected 1889 question–answer texts from the most active online health community in China, Good Doctor Online, and used these public data to identify five types of entities. In addition, we conducted a comparative evaluation with three other commonly used models to validate the performance of our proposed model, including RoBERTa-CRF (RC), BilSTM-CRF (BC), and RoBERTa-Softmax (RS). The results showed that the RBC model achieved excellent performance on the test set, with an accuracy of 81.2% and an F1 score of 80.7%, outperforming the performance of traditional entity recognition models in named entity recognition in online medical communities for doctors and diabetes patients. The high performance of entity recognition in online health communities will provide a crucial knowledge source for constructing medical knowledge graphs. This integration would help alleviate the growing demand for medical consultations and the strain on healthcare resources, while assisting healthcare professionals in making informed decisions and providing personalized services to patients.

## 1. Introduction

In 2030, it is expected that 11.3% of adults will have diabetes, which would affect roughly 643 million people. Diabetes is one of the most rapidly expanding global crises of the 21st century [1]. Relevant studies have indicated that roughly half of web-based health information users with chronic health issues may benefit from accessing online health information [2]. The Q&A structure of online health communities (OHCs) is becoming more and more popular, with diabetic patients seeking medical knowledge and diabetic self-management assistance [3,4,5].Online health communities store a significant amount of case information, medical knowledge, and prescription data, which serves as the hotspots for medical big data applications.

In the doctor–patient Q&A texts from the online health community, entities can be identified as linked to diseases, medications, tests, treatments, and symptoms for diabetes patients, and used to provide various intelligent services to diabetic patients. We can also gain a deeper understanding of patients’ needs and interests in health-related information through entity recognition [6].With this knowledge mined from online health communities, we can then offer patients individualized medical care, health information, decision-making participation, emotional support services, and improvements in online medical services.

Historically, vast sets of rules or lexicons had to be manually created by professionals for both rule-based and lexicon approaches to medical entity recognition [7,8,9]. Using benchmark data from the i2b2 2009 drug challenge and a hybrid lexicon-based and rule-based model, [10] achieved an F1 score of 66.97% for the named entity recognition of pharmaceuticals. Statistics-based machine learning algorithms leveraging manually annotated corpora for supervised training have exhibited a significant increase in accuracy over rule-based and lexicon-based entity recognition approaches [11,12]. With the advent of deep learning, numerous neural-network-based models have effectively been used for the textual entity recognition of biological documents [13,14], electronic medical records [15,16,17], and online health communities [18,19,20] Dreyfus Dreyfus. Based on the entity recognition infrastructure deep learning model LSTM-CRF, Guillaume Lample et al. [21] proposed a neural network model that combines bidirectional long short-term memory (BiLSTM) and conditional random fields (CRFs). This bidirectional structure enables the capture of sequential information in context, leading to widespread applications in entity recognition. Wang, Z. et al. [22] retrieved input patient fundamental information and illness information, annotated entities on medical community Q&A texts, and trained a BiLSTM-CRF to recognize and extract entities linked to diabetes in the medical domain. However, the BiLSTM-CRF model focuses on extracting features between words and characters from the text while disregarding the contextual meaning of context. To address this issue, Jacob Devlin et al. from Google introduced a BERT pre-training model [23]. This model improved the quality of embedding words and reduced the workload of downstream classification tasks, resulting in better recognition performance. In recent years, named entity identification in electronic medical records [24,25,26] and biomedical literature [27] has been successfully implemented with BERT, a pre-trained model with enhanced contextual long-range semantic learning capability based on word vectors.

Due to their lack of medical knowledge, users of the online healthcare communities for diabetes produce texts that contain inaccurate or slang expressions. Entity recognition of Q&A text in online health communities is challenged with semantic ambiguity, content heterogeneity, high complexity, and imperfect recognition; hence, it is difficult to achieve the desired outcome. Some studies have shown that applying the RoBERTa model to named entity recognition tasks improves the entity recognition performance (F1 score) [28]. To mitigate the impact of Chinese online health data on the performance of entity recognition, this paper utilizes a combined model of RoBERTa-BiLSTM-CRF to accomplish medical entity recognition tasks related to diabetes. This method primarily addressed the following tasks: (1) We standardized the diabetes annotation corpus of the online health community using the diabetes entity classification standards of Ruijin Hospital; (2) The pre-trained model RoBERTa-BiLSTM-CRF was used to identify named entities in Q&A text from the Good Doctor Online health community, and evaluated by comparing it with the other three models; (3) The entity recognition performance of the Q&A texts from the perspective of the patient was compared with that of electronic medical records from the clinician’s perspective.

## 2. Method

### 2.1. Data Collection and Preprocessing

**Figure 1:**
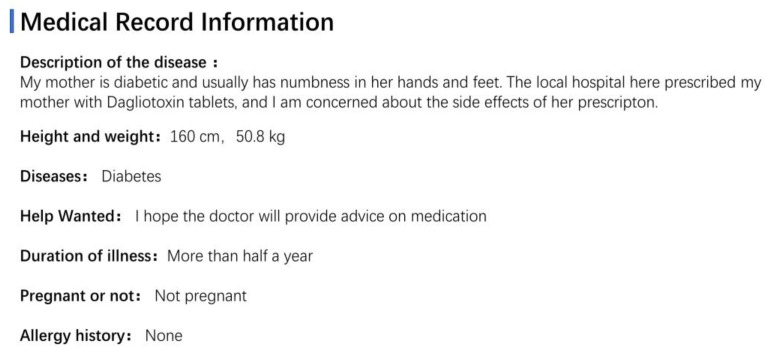
Online doctor-patient Q&A text structure (from the website’s original screenshot).

We chose the top Chinese online doctor–patient Q&A platform, “Good Doctor Online” (https://www.haodf.com/, accessed on 5 December 2021), searched the Q&A section of the diabetes-specific disease section, and collected 9446 questions from November 2020 to November 2021. When consulting doctors, patients submitted content using a specified information description framework, as shown in Figure 1. 

**Figure 2:**
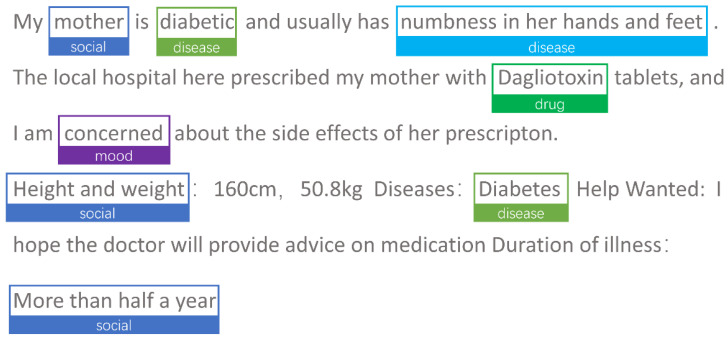
Annotation tool interface (from the website’s original screenshot).

**Figure 3:**
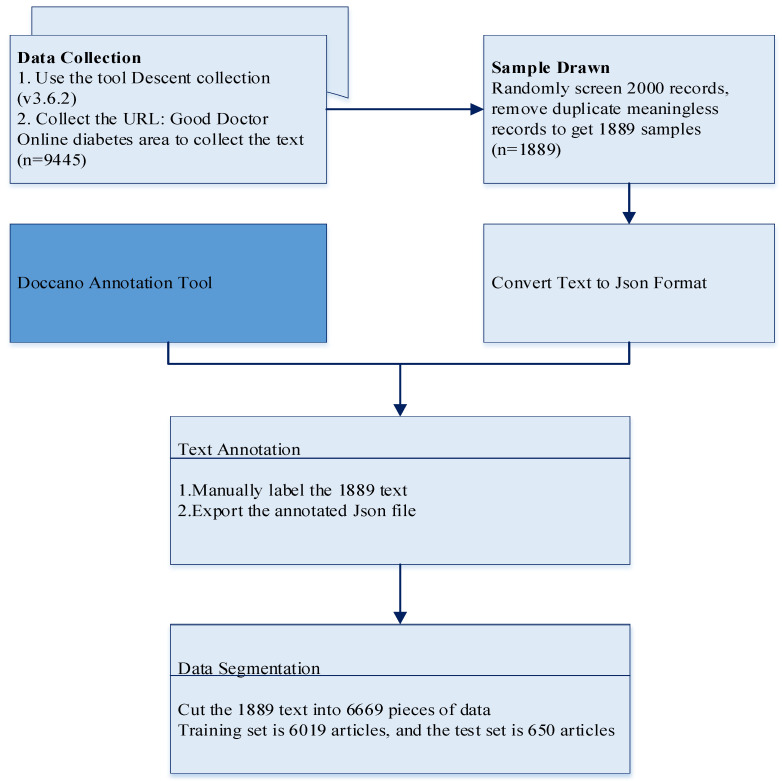
Data preprocessing flow chart.

For this study, the following preprocessing processes were carried out: (1) Removed all non-textual content (replacing emoji icons with emoji-related codes); (2) Filtered 2000 values at random from the acquired dataset of 9445 values, deleted duplicate and nonsensical data to obtain 1889 values, and converted the data to JSON format; (3) Annotated the questions of health community Q&A text into eight categories (check, disease, drug, mood, life, social, symptom, and treat) using the Doccano annotation tool. Figure 2 depicts the annotation interface; (4) To process the exported text, it was divided into 6669 values. The dataset was then further split into a training set consisting of 6019 data slices and a test set consisting of 650 data slices. The ratio of this split was approximately 9:1. Within the training set, the data were divided into a training subset and a validation subset, at a ratio of 5:1. Next, we converted the JSON format files into a data format for generic named entity recognition tasks using BIO tagging; (5) Utilized the RoBERTa word vector model made available by the Harbin Institute of Technology as an open source. Figure 3 depicts the specific data preprocessing procedure.

### 2.2. RoBERTa-BiLSTM-CRF Model Construction

**Figure 4:**
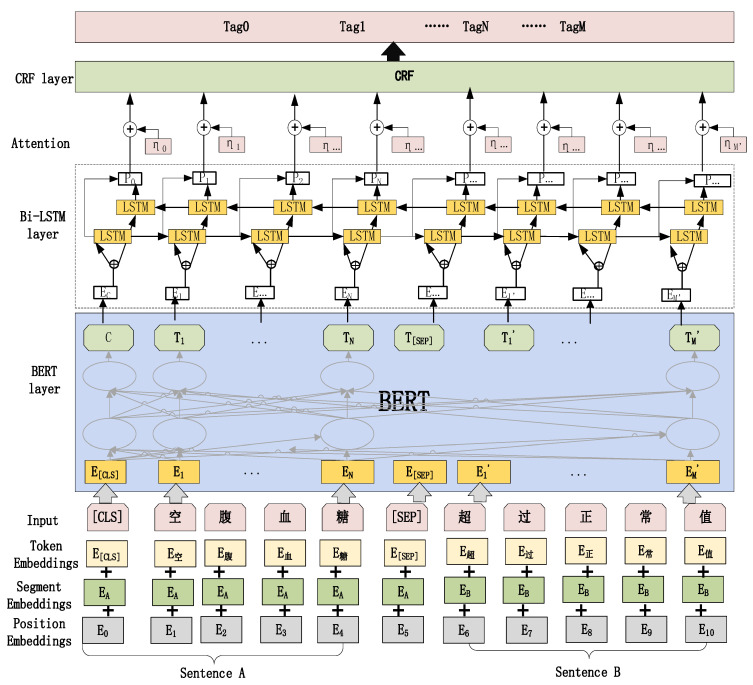
Structural diagram of the Bert-BilSTM-CRF model.

This article employed the RoBERTa-BiLSTM-CRF model, which is composed of three layers: the RoBERTa word vector layer, the BiLSTM layer, and the CRF layer. In the word vector layer, word embedding and model construction were carried out by applying the Chinese pre-training model from the HUST Xunfei Lab in order to obtain word-level vector information and a semantic representation suitable for the Chinese language. The BiLSTM layer is utilized for semantic encoding, and forward and backward LSTM networks are used for each training sequence; the forward and backward networks were connected to the same output layer. The CRF layer, which effectively evaluated the labeling information before and after the sequence, filtered out entities that did not conform to the labeling rules and outputs a sequence with the best likelihood of being correctly categorized. Figure 4 depicts the general structure of the RoBERTa-BiLSTM-CRF model.

#### 2.2.1. RoBERTa Pre-Training Layer to Construct Word Vectors

Each input word of the encoder generated three vectors, denoted by vectors, accordingly. After calculating the inner product between and producing the similarity weights, the similarity was calculated. Then, the weights were normalized to a value between 0 and 1, and the similarity vector was processed using the function shown in Equation (1).


(1) $$\alpha_{i}=softmax\left(f\left(Q,K_{i}\right)\right)=\frac{exp\left(f\left(Q,K_{i}\right)\right)}{\sum_{i}exp(f(Q,K_{i}))}$$

Scaling was accomplished by multiplying $\frac{1}{\sqrt{d_{k}}}$ with the result of the inner product of Q and K. The attentional mechanism is presented in Equation (2).

(2) $$Attention\left(Q,K,V\right)=softmax\left(\frac{QK^{T}}{\sqrt{d_{k}}}\right)V$$

Combining the outcomes of attention processes yielded the multi-headed attention module, as determined using Equation (3).

(3) $$MultiHead\left(Q,K,V\right)=Concat\left(head_{1},head_{2},head_{3},\ldots ,\;head_{h}\right)W^{o}$$

The output of the multi-headed attention layer was then passed to the feed-forward neural network, the module described in Equation (4).

(4) $$FFN\left(Z\right)=max\left(0,ZW_{1}+b_{1}\right)W_{2}+b_{2}$$

The output layer employed a self-supervised approach to estimate the probability that the masked target word and the two phrases shared a contextual link. After multiple training iterations, the likelihood and the weight parameter with the largest value for the two tasks are determined.

#### 2.2.2. Layer of BiLSTM for Semantic Encoding

Long short-term memory networks incorporate memory units in the hidden layer, which can better solve the problem of gradient disappearance caused by excessively long sequences in the training of conventional recurrent neural networks, enabling them to be more effectively used in the named entity recognition task. Its structure consists of the following equations:

(5) $$i_{t}=\sigma \left(x_{t}\cdot w_{xh}^{i}+h_{t-1}\cdot w_{hh^{\prime }}^{i}+b_{h}^{i}\right)$$

(6) $$f_{t}=\sigma \left(x_{t}\cdot w_{xh}^{f}+h_{t-1}\cdot w_{hh^{\prime }}^{f}+b_{h}^{f}\right)$$

(7) $$o_{t}=\sigma \left(x_{t}\cdot w_{xh}^{o}+h_{t-1}\cdot w_{hh^{\prime }}^{o}+b_{h}^{o}\right)$$

(8) $$c\prime_{t}=\tanh\left(x_{t}\cdot w_{xh}^{c}+h_{t-1}\cdot w_{hh^{\prime }}^{c}+b_{h}^{c}\right)$$

(9) $$c_{t}=i_{t}⊗{c^{\prime }}_{t}+f_{t}⊗{c^{\prime }}_{t-1}$$

(10) $$h_{t}=o_{t}⊗\tanh\left(c_{t}\right)$$

The σ denotes the Sigmoid activation function, ⊗ is the dot product operation, and $x_{t}$ is used as the unit input; $i_{t}$,$f_{t}$,$o_{t}$ denotes the input gate, forgetting gate, and output gate at a specific moment, respectively; tanh denotes the hyperbolic tangent activation function; w,b represent the weight matrix and bias vector of the input gate, forgetting gate, and output gate, respectively; $c{’}_{t}$ represents the state at time, which is the intermediate state obtained only from the current input and is used to update the state at time t; $h_{t}$ represents the output at time t.

The BiLSTM bi-directional long and short-term memory network with forward and reverse LSTM for each word sequence was used to decode the text sentences in the input layer, and data conversion and transfer through forward LSTM and backward LSTM were used to acquire contextual feature vectors in both directions. First, the output calculated the error existing in the output layer at each moment, followed by the derivatives of parameters of the forward LSTM from moment t to moment 1. For the network portion of the backward LSTM, loss needs to be calculated from moment 1 to moment t, and reverse differentiation be conducted. The formula for the output is provided in the following equations:

(11) $${\vec{h}}_{t}=LSTM_{L}\left({\vec{x}}_{t},{\vec{h}}_{t-1}\right)\;$$

(12) $${\overset{↼}{h}}_{t}=LSTM_{R}\left({\overset{↼}{x}}_{t},{\overset{↼}{h}}_{t-1}\right)$$

(13) $$\;h_{t}=\left[{\vec{h}}_{t},{\overset{↼}{h}}_{t}\right]\;$$

#### 2.2.3. CRF Optimized Tag Sequence

CRFs can compensate for the shortcomings of BiLSTM by providing an ideal sequence of predictions based on the relationship between surrounding labels. The output score matrix of BiLSTM is supposed to be P for any arbitrary sequence $X=\left(x_{1},x_{2},\ldots ,x_{n}\right)$. The size of P is n×k, where n represents the number of words, k represents the number of tags, and $P_{ij}\;$ represents the score of the jth tag of the word. Equation (14) describes the score function for the sequence of predictions $Y=\left(y_{1},y_{2},\ldots ,y_{n}\right)$.

(14) $$s\left(X,Y\right)=\sum_{i=0}^{n}Ay_{i},y_{i+1}+\sum_{i=0}^{n}P_{i},y_{i}$$

A denotes the matrix of transferred scores, $A_{ij}$ represents the scores transferred from label i to label j, and the size of A is k+2. Equation (15) describes the probability of generating the predicted sequence Y.

(15) $$p\left(Y|X\right)=\frac{e^{s\left(X,Y\right)}}{\sum_{\tilde{Y}\in Y_{X}}s\left(X,\tilde{Y}\right)}$$

The probability function of the expected sequence could be obtained by taking the logarithm at both ends.


(16) $$\ln\left(p\left(Y|X\right)\right)=s\left(X,Y\right)-\ln(\sum_{\tilde{Y}\in Y_{X}}s\left(X,\tilde{Y}\right))$$

In Equation (17), $\tilde{Y}$ denotes the true labeled sequence, whereas $Y_{X}$ denotes all conceivable labeled sequences. Decoding yielded the output sequence corresponding to the maximum score.

(17) $$Y^{*}=\underset{\tilde{Y}\epsilon Y_{X}}{\arg maxs(X,\tilde{Y})}$$

## 3. Result

### 3.1. Text Annotation

Health Community Q&A texts are self-reported by patients to their physicians; therefore, the language of the text differed from that of the medical literature and electronic medical records. When annotating, it is important to note the frequent abbreviations and misspellings. The original words were precisely aligned with the common words. Under the supervision of two medical informatics professionals and one medical expert, we coded each record in terms of the classification criteria for diabetes mellitus at Ruijin Hospital. This labeling was divided into eight categories (check, disease, drug, lifestyle, mood, social context, symptom, and treatment). Table 1 summarizes the classification criteria.

### 3.2. Experimental Setup

This study was based on the Python + PyTorch + GPU deep neural network learning framework. The cross-entropy loss function was used as the loss function, and the AdamW method was employed for model training optimization. A five-fold cross-validation procedure was utilized to run our proposed model. During the training process, we performed fine-tuning on Roberta. The input dimension, sequence_length, was set to 128. The initial learning rate of the model was set to 3 × 10^−5^, while the learning rates of BiLSTM and CRF were set to five times greater than that of Roberta, namely, 1.5 × 10^−4^. A cosine schedule with a warmup was used to adjust the learning rate. We set the warmup steps to one-tenth of the total training epochs, and the learning rate decay rate was set to 0.01 (weight_decay). The word embedding dimension (pooler_fc_size) was set to 768, and the batch size was set to 16. Dropout was applied with rates of 0.1 at the input layer and hidden layers. The total number of training epochs was set to 50, and the F1 score was calculated on the validation set after each epoch. The best model was saved accordingly. The patient number was set to 10, which means that if the model did not show improvement on the validation set over 10 consecutive epochs, the training would be terminated early. The weights, biases, and other parameters were continuously optimized during the training process. To prevent issues such as gradient explosion or vanishing gradients during code execution, the gradient clipping technique was employed. The performance of the best model was tested on the final test set, which was not used during model training. The F1 score was calculated for each category, and the average score was taken as the F1 score on the test set. The average F1 score from the five rounds of cross-validation was calculated as the final F1 score. The experimental parameters for model training are summarized in Table 2.

### 3.3. Evaluation

**Figure 5:**
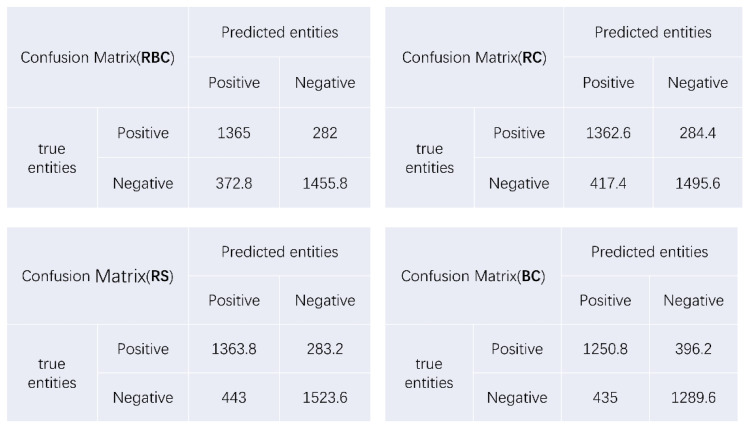
Confusion matrix for the four models.

This study examined the performance of the model by calculating its precision, recall, accuracy, and F1 scores. TP, TN, FP, and FN are the number of positive samples correctly predicted for the positive class, the number of samples correctly predicted for the negative class, the number of samples incorrectly predicted to be in the positive class, and the number of samples incorrectly predicted to be in the negative category, respectively. In this study, the entity array obtained through manual annotation was referred to as the truth entity set, while the array of entities predicted by the machine learning model after training was called the predicted entity set. Taking the intersection of these two arrays, the number of entities that appear in both arrays was defined as true positives (TPs), indicating that the machine successfully predicted the true entities. The number of entities in the truth entity set that were not correctly predicted was defined as false negatives (FNs), while the portion of entities in the predicted entity set that were not correctly predicted was defined as false positives (FPs). Figure 5 presents the confusion matrices for the four models.

(18) $$Pre=\frac{TP}{TP+FP}$$

(19) $$Re=\frac{TP}{TP+FN}$$

(20) $$F1=\frac{2\times Pre\times Re}{Pre+Re}$$

(21) $$Accuracy=\frac{TP+TN}{TP+TN+FP+FN}$$

Precision refers to the ratio of actual positive samples to expected positive samples. Recall, also known as sensitivity, is the percentage of predicted true-positive samples to the total number of true-positive samples. The F1 value is a combined precision and recall rating. Accuracy reflects a model’s ability to correctly classify the overall samples, i.e., the proportion of samples that are correctly predicted among all samples.

### 3.4. Model Performance

To verify the validity and feasibility of the model, the total experimental results of our RBC model and other excellent models are shown in Table 3; the baseline model was BC. Other models are RC and RS. The experimental results demonstrate that our suggested RBC model enhanced precision by 4.3%, recall by 7%, F1 values by 5.6%, and Acc values by 5.8%, and had a better overall performance when compared with the BiLST-CRF baseline model. We used five-fold cross-validation, training, and testing on the corpus; the final results are shown in Table 3.

Table 4 presents a statistical evaluation of the effectiveness of eight distinct entity recognition categories. We observed that two entity types, emotional and social attributes, achieved superior results with significantly higher precision, recall, and F1 values than other entity types, whereas two entity types, symptoms and therapies, were significantly less effective. For the identical CRF model based on words, the RBC and RC impacts were extremely similar.

We identified eight entity types from Q&A texts: check, disease, drug, lifestyle, emotion, social attribute, symptom, and treatment. Table 5 displays the distribution of the eight types of entities in the 1890 records. The entities with the highest frequency were social properties, diseases, and tests, which accounted for 89.68, 80.05, and 80.00%, respectively; followed by drugs, symptoms, lifestyle, and treatment, which accounted for 56.4, 36.40, and 25.93%, respectively. The less frequent entities were symptoms and emotions, which accounted for 7.61 and 7.59%, respectively. In addition, we counted the top 10 highest-frequency words of each entity type. For example, among 4259 check entity types, fasting blood glucose, postprandial blood glucose, glycated hemoglobin, and glucose tolerance tests were the most common tests for diabetes; these high-frequency words accounted for 70.86% of the examination entity categories. Among the disease entity types, hypertension, fatty liver, coronary heart disease, cerebral infarction, and stroke were the most frequently occurring diseases; these high-frequency words accounted for 73.15% of the disease entity categories, indicating that diabetic patients are often afflicted by other types of cardiovascular diseases and complications. Table 5 describes the details of the top 10 entities.

## 4. Discussion

Combining the diabetes entity classification criteria of Shanghai Ruijin Hospital, our model demonstrated that the RoBERTa-BiLSTM-CRF-based deep learning model could perform the online Q&A text-based diabetes entity recognition task with an F1 value of 81.51%, outperforming previously published online healthcare entity recognition results using the BiLSTM-CRF model (68.43%) [29]. This is comparable to the recently reported BERT-BiLSTM-CRF-model-based named entity recognition system for the diabetes literature (79.89%) [30]. The benefit of the RoBERTa-BiLSTM-CRF model (F1 value of 81.51%) over the benchmark model, BiLSTM-CRF (F1 value of 75.28%), is that BERT produces better word-level vectors than the phrase vectors acquired using Word2vec. Pre-training in the biomedical corpus improves BERT’s ability to comprehend difficult biomedical literature.

The semi-structured doctor–patient health community requires patients to fill in socio-demographic data and provides optional fixed-word input, which may indicate that socio-demographic information descriptions are relatively standardized and fixed, and the accuracy and sensitivity of entity recognition were improved, with F1 values exceeding 90% for all four models. In addition, the patients’ inputs in the text boxes of “chief complaint” and “help wanted” were relatively free text, and the majority of patients used colloquial language to describe their symptoms and treatments due to a lack of professional knowledge. The “Help” text box contained a highly free-form description written primarily in colloquial language, with a certain number of misspellings and ambiguities regarding the concept of professional terms, which are significantly different from the electronic medical records portrayed from the physician’s perspective. The language style of the doctor–patient Q&A community is information-oriented language expression, which is characterized by specific, certain, and objective vocabularies, while the language style of the patient–patient community is social-support-oriented language expression, characterized by ambiguous and empathic features. In the Chinese electronic medical record dataset, CCKS, based on the BERT model, published studies demonstrating that the F1 values for the symptom-sign category all exceeded 95% and the F1 values for the treatment entity category all exceeded 82% [31,32]. Additionally, the entity recognition was superior to the entity recognition in online health communities [33,34]. It has been demonstrated that biomedical experts and the general public differently perceive medical entities in diabetes.

In addition, we analyzed named entities extracted from online health communities to investigate the key topics discussed and emphasized in patients’ online health Q&As for the purpose of studying the health information needs of patients. Table 5 shows the frequency of entity occurrences in each category and the proportion of TOP10 entities in the respective entity type. The frequency indicates the number of times a category of entities is mentioned in relevant posts. In 1890 relevant posts, for example, the test and examination category entities were mentioned 1512 times. The experimental data suggest that they focus on diseases (possibly assessment screening for diabetes and complications of diabetes), tests and examinations (on diabetes screening and concerns about glycemic control management), and medications (possibly counseling on medication involving diabetes), as confirmed by previously published studies on entity identification in online health communities for diabetes [35,36].

The limitations of this study include the following: (1) The data sources only comprised single online health community doctor–patient Q&A texts, without considering differences in recognition performance of the BERT model on datasets from other online health communities with different language styles. Furthermore, the study lacked a comprehensive investigation into the connection between language expression features of different chronic diseases and the applicability of the chosen model. Therefore, further research must be conducted on the applicability of our model to online health community texts. (2) The diabetes data used in the study were cross-sectional static data of patients, and longitudinal cohorts of different stages of chronic disease progression were not collected without patient tracking [37]. Future research must continue to standardize the annotated corpus, expand its coverage, and optimize the outcomes of the model.

## 5. Conclusions

For the named entity recognition of the online medical community of diabetes, the RoBERTa-LiSTM-CRF model outperforms the other three models: RoBERTa-CRF (RC), BilSTM-CRF (BC), and RoBERTa-Softmax (RS). The proposed model, consisting of a pre-trained model with enhanced contextual long-range semantic learning ability based on word vectors, can effectively address entity recognition challenges within the health community. In addition, we found that patients with different disease stages have distinct focused topics and that the extracted entity type and attribute values will also vary. The high-performance entity recognition in online health communities represents a crucial knowledge source for constructing medical knowledge graphs. It can be applied to intelligent question-answering systems, clinical decision support systems, and other applications. This integration helps alleviate the growing demand for medical consultations and the strain on healthcare resources while assisting healthcare professionals in making informed decisions and providing personalized services to patients. In our future research, we will implement the BERT model for pre-training on additional websites of online healthcare communities.

## Figures and Tables

**Table 1:** Labeling classification standards.

Classification	Description	Labeling Case
Check	Test and examination items, physical examination, review, etc.	A review at the hospital the previous day; a check-up at the hospital.
Disease	Disease names, such as hypertension, diabetes, etc.	No diabetes in the family either.
Drug	The name of the drug, such as nifedipine, metformin, nifedipine, etc.	The medications being taken are Metformin Hydrochloride and Vildagliptin.
Lifestyle	Patient’s lifestyle, e.g., smoking, alcohol consumption, sleep, etc.	Smoking; drinking; staying up late.
Mood	Irritable, anxious, worried	So now it is confusing.
Social context	Dad (my dad), wife (my wife), medical history, occupation, height, weight, age, gender (pregnancy and gestation), wanting children.	Height and weight: 171 cm, 70 kg. Pregnancy: not pregnant.
Symptom	Patient’s subjective description of feelings and signs (skin jaundice), such as dizziness, non-dizziness, nocturia, puffy eyelids, and frequent need to urinate.	Feeling of vertigo when standing suddenly; I urinate frequently and often, but each time the amount of urine is not much, nausea, vomiting, weakness, stomach pain, and breast swelling.
Treatment	Chinese medicine treatment, immunotherapy, ventilator, and stent release.	Immunotherapy.

**Table 2:** Experimental parameters.

Experimental Parameters	Value
Sequence_length	128
Batch_size	Train set 16, test set 16
Pooler_fc_size	768
Epoch	50
Learning rate	3 × 10^−5^
Optimizer	Adam
Input layer dropout	0.1
Hidden layers dropout	0.1

**Table 3:** Comparative experimental results of four models on the test set.

Models	F1	P	R	Acc
RBC	0.807	0.786	0.829	0.812
RC	0.795	0.755	0.827	0.803
RS	0.790	0.755	0.828	0.799
BC	0.751	0.743	0.759	0.754

**Table 4:** Evaluation of the effect of different entity recognition of four models.

Model	Index	Check	Disease	Drug	Lifestyle	Mood	Social	Symptoms	Treat
RBC	P	0.739	0.787	0.730	0.754	0.865	0.941	0.609	0.571
	R	0.774	0.863	0.823	0.723	0.922	0.926	0.709	0.585
	F1	0.756	0.823	0.774	0.738	0.892	0.933	0.655	0.578
RC	P	0.719	0.761	0.730	0.696	0.903	0.918	0.596	0.542
	R	0.771	0.850	0.821	0.732	0.933	0.924	0.710	0.639
	F1	0.744	0.803	0.773	0.713	0.917	0.921	0.647	0.586
RS	P	0.717	0.742	0.704	0.697	0.878	0.915	0.582	0.516
	R	0.772	0.866	0.824	0.726	0.956	0.923	0.690	0.624
	F1	0.743	0.799	0.759	0.710	0.915	0.919	0.631	0.564
BC	P	0.682	0.722	0.697	0.702	0.857	0.918	0.533	0.559
	R	0.731	0.766	0.684	0.668	0.756	0.909	0.521	0.610
	F1	0.704	0.748	0.687	0.681	0.795	0.913	0.526	0.582

**Table 5:** Related statistics of entity frequency.

Entity Type	Entity Frequency	Rate	Top 10 Entities	Top 10 Number of Entities	Top 10 Rate
Check	1512/1890	80%	Blood glucose, fasting blood glucose, fasting, postprandial, glycated hemoglobin, physical examination, high blood glucose, glucose tolerance, review, and postprandial blood glucose.	3019/4259	70.86%
Disease	1513/1890	80.05%	Diabetes, hypertension, hyperglycemia, type 2 diabetes, fatty liver, coronary heart disease, cerebral infarction, obesity, hyperlipidemia, and complications of diabetes.	1790/2447	73.15%
Drug	1066/1890	56.4%	Insulin, Metformin, Acarbose, Glucose, Dapagliflozin, Glucagon, Glimepiride, Bystolic, Gleevec, and Chinese medicine.	1306/2504	52.16%
Life	490/1890	25.92%	Blood sugar control, exercise, diet control, poor sleep, stopping the medication, exercise, not taking medication, losing weight, watching what you eat, and staying up late.	509/787	64.68%
Mood	144/1890	7.61%	Worry, doubt, fear, anxiety, hurry, tension, tiredness, anger, uneasiness, and fear.	132/185	71.35%
Social	1695/1890	89.68%	Height and weight, greater than six months, pregnant, not pregnant, within six months, within one month, within one week, self, allergy, and father.	3864/4690	82.39%
Symptom	688/1890	16.4%	Thirst, bitterness and dryness, dizziness and lightheadedness, excessive urination, weakness, weight loss, nausea, sweating, panic attacks, and frequent urination.	465/1564	29.73%
Treat	421/1890	22.08%	Surgery, chemotherapy, radiotherapy, drug therapy, inpatient treatment, weight loss, Chinese medicine, stents, minimally invasive, and immunization.	429/655	65.50%

## Data Availability

The datasets used and/or analyzed during the current study are available from the corresponding author upon approved written requests.
